# Soluble LRIG2 Ectodomain Is Released from Glioblastoma Cells and Promotes the Proliferation and Inhibits the Apoptosis of Glioblastoma Cells *In Vitro* and *In Vivo* in a Similar Manner to the Full-Length LRIG2

**DOI:** 10.1371/journal.pone.0111419

**Published:** 2014-10-29

**Authors:** Qungen Xiao, Yihu Tan, Yang Guo, Hongkuan Yang, Feng Mao, Ruifan Xie, Baofeng Wang, Ting Lei, Dongsheng Guo

**Affiliations:** Department of Neurosurgery and Sino-German Neuro-Oncology Molecular Laboratory, Tongji Hospital, Tongji Medical College, Huazhong University of Science and Technology, Wuhan, P.R. China; University of Udine, Italy

## Abstract

The human leucine-rich repeats and immunoglobulin-like domains (LRIG) gene family contains LRIG1, 2 and 3, encoding integral membrane proteins with an ectodomain, a transmembrane domain and a cytoplasmic tail. LRIG1 negatively regulates multiple receptor tyrosine kinases signaling including the epidermal growth factor receptor (EGFR) and is a proposed tumor suppressor. The soluble LRIG1 ectodomain is demonstrated to be shed naturally and inhibit the progression of glioma. However, little is known regarding the functions of LRIG2. In oligodendroglioma, LRIG2 expression is associated with poor survival, suggesting that LRIG2 might have different functions compared with LRIG1. Since soluble LRIG1 ectodomain has a similar function to the full-length LRIG1, we hypothesize that the different roles exerted by LRIG2 and LRIG1 result from the difference of their ectodomains. Here, we addressed the functions of LRIG2 and LRIG2 ectodomain in the proliferation and apoptosis of glioma and the possible underlying mechanisms. Firstly, we found that LRIG2 expression levels positively correlated with the grade of glioma. Further, we demonstrated for the first time that soluble LRIG2 ectodomain was capable of being released from glioblastoma cells and exerted a pro-proliferative effect. Overexpression of LRIG2 ectodomain promoted the proliferation and inhibited the apoptosis of glioblastoma cells *in vitro* and *in vivo* in a similar manner to the full-length LRIG2. Both full-length LRIG2 and LRIG2 ectodomain were found to physically interact with EGFR, enhance the activation of EGFR and its downstream PI3 K/Akt pathway. To our knowledge, this is the first report demonstrating that soluble LRIG2 ectodomain is capable of being released from glioblastoma cells and exerts a similar role to the full-length LRIG2 in the regulation of EGFR signaling in the progression of glioblastoma. LRIG2 ectodomain, with potent pro-tumor effects, holds promise for providing a new therapeutic target for the treatment of glioblastoma.

## Introduction

Glioblastoma multiforme (GBM) is by far the most common and lethal type of brain cancer. Despite the recent improvements in surgery, radiation therapy and cytotoxic chemotherapy, the prognosis for GBM remains grim, with a median survival time of only 12–15 months after diagnosis [1]. Thus, the development of novel efficacious therapies is greatly warranted to improve the poor prognosis of patients afflicted with GBM.

Substantial research effort has focused on the identification of genetic alterations in GBMs that might help response to specific therapies. The most common genetic alteration associated with GBM is the amplification of the epidermal growth factor receptor (EGFR), with a frequency of about 50% [2].The ligand-binding triggered the activation of amplified EGFR, resulting in enhanced downstream signaling controlling pleiotropic cellular responses, such as cell proliferation and survival [3]. Owing to the vital role of the EGFR activation in glioblastoma progression, the understanding of its endogenous regulators has been a subject of intense interest.

In the research on the negative regulators of EGFR, the human leucine-rich repeats and immunoglobulin-like domains (LRIG) gene family was found [4]. The mammalian LRIG gene family is composed of three paralogous genes, namely LRIG1, LRIG2 and LRIG3, which encode integral membrane proteins, with a signal peptide, an extracellular part consisting of 15 leucine-rich repeats (LRR) with cysteine-rich N- and C-terminal flanking domains and three immunoglobulin-like domains, followed by a transmembrane domain and a cytoplasmic tail [4]. LRIG1, the best-studied LRIG family member, negatively regulates the signaling pathways mediated by ERBB [5], [6], MET [7] and RET [8] receptor tyrosine kinases, and is suggested to be a tumor suppressor [9]. LRIG1 is down-regulated and associated with a favorable prognosis in many cancers [10], [11], [12], [13]. Inhibition of EGFR signaling by LRIG1 results from a physical interaction between the extracellular domain of both proteins, inducing the recruitment of E3 ubiquitin ligases, follow by internalization and enhanced lysosomal degradation of the protein complex [5], [6]. Recently, soluble LRIG1 ectodomain is demonstrated to be released naturally by proteolytic shedding and suppress EGF signaling without any apparent EGFR protein downregulation [14]. Moreover, soluble extracellular part of mouse Lrig1 is capable of inhibiting glioma growth *in vitro* and *in vivo* irrespective of EGFR status [15]. LRIG3 appears to have a similar role to LRIG1 in the progression of glioma [16], [17], [18]. However, little is known regarding the molecular and developmental functions of mammalian LRIG2. Recently, it was found that Lrig2-deficient mice were protected against PDGFB-induced glioma [19]. In addition, LRIG2 expression is associated with poor survival in oligodendroglioma [20] and squamous cell carcinoma of the uterine cervix [21]. Noteworthy, we previously demonstrate that downregulation of LRIG2 inhibits glioblastoma cell growth in *vitro* [22]. To date, the data suggest that LRIG2 might promote the genesis of tumors and have different, possibly opposing, functions compared with LRIG1. The mechanism underlying the different roles played by LRIG2 and LRIG1 in the progression of tumors has not been explored yet. Based on the findings that soluble LRIG1 ectodomain has the similar functions to the full-length LRIG1 and full-length LRIG2 plays different roles compared to the full-length LRIG1, it is reasonable to hypothesize that the LRIG2 ectodomain may give rise to the special role played by LRIG2.

In this study, we studied the functions of the full-length LRIG2 and LRIG2 ectodomain in the progression of glioblastoma to gain insight into the mechanism underlying the role of LRIG2. We firstly investigated the relationship between the expression level of LRIG2 and the grade of glioma. Further, we generated glioblastoma cells with stable expressions of the full-length LRIG2 and LRIG2 ectodomain and investigated the effects of full-length LRIG2 and LRIG2 ectodomain on the proliferation and apoptosis of glioblastoma *in vitro* and *in vivo.* We then explored the possible mechanisms underlying the effects. Strikingly, we demonstrated for the first time that the soluble LRIG2 ectodomain was capable of being secreted by glioblastoma cells and exerted a pro-proliferative effect. Both full-length LRIG2 and LRIG2 ectodomain promoted the proliferation and inhibited the apoptosis of glioblastoma *in vitro* and *in vivo* probably through enhancing the EGFR activation and its downstream PI3K/Akt pathway. To our knowledge, this is the first report showing that the soluble LRIG2 ectodomain, which can be released from glioblastoma cells, positively regulates the growth of glioblastoma and EGFR-mediated PI3 K/Akt signaling in a similar manner to the full-length LRIG2.

## Materials and Methods

### TCGA Data and Glioma Sample Description

For expression analysis according to WHO grade, gene expression data of glioblastoma multiforme (GBM) and brain low-grade glioma (LGG) were downloaded from the public TCGA data repositories (https://tcga-data.nci.nih.gov/tcga/tcgaDownload. jsp) (date of download: December 2013), which include 27 cases of LGG and 466 cases of GBM. Fresh clinical glioma samples of different grade (20 cases of LGGs and 20 cases of high-grade gliomas (HGGs)) were obtained from the Department of Neurosurgery, Tongji Hospital after informed consent from the patients according to the Declaration of Helsinki. Diagnosis and grading was according to the WHO 2007 criteria [23]. The collection and usage of patient tumor tissue samples was approved by the appropriate local ethics committees and the consents of all the patients were written and approved (Institutional Review Board, Tongji Hospital, Tongji Medical College, Huazhong University of Science and Technology, IRB ID: 20121202).

### Antibodies and Reagents

The mouse anti-Flag monoclonal antibody (Sigma F1804) was purchased from Sigma-Aldrich (St.Louis, MO, USA). The Ki-67 (Abcam ab16667) and PCNA (Abcam ab92552) rabbit monoclonal antibodies were purchased from Abcam (Cambridge Science Park) and the anti-LRIG2 rabbit polyclonal antibody was generously provided by Prof. Håkan Hedman (Umeå University, Sweden). The following antibodies used in western blotting were diluted in TBST/5%BSA: Anti-EGFR 1∶1000 (Cell signaling 4267), anti-pEGFR 1∶1000 (Cell signaling 2220), anti-Akt 1∶1000 (Cell signaling 9272), anti-pAkt 1∶1000 (Cell signaling 4058), anti-cMyc 1∶1000 (Cell signaling 5605), anti-cyclin B1 1∶1000 (Cell signaling 4138), anti-cyclin D1 1∶1000 (Cell signaling 2978), anti-cyclin E 1∶200 (Santa Cruz, sc-481), anti-Bcl-2 1∶1000 (Cell signaling 2870), anti-Bax 1∶1000 (Cell signaling 5023), and anti-caspase-3 1∶1000 (Cell signaling 9662). Mitochondrial membrane potential assay kit with JC-1 was purchased from Beyotime Institute of Biotechnology (Jiangsu, China).

### Cell Lines and Cell Culture

The U87 and U251 human glioblastoma cell lines, purchased from American Type Culture Collection, were cultured in Dulbecco’s modified Eagle’s medium (DMEM) supplemented with 10% (v/v) fetal bovine serum (FBS) (Hyclone, USA) in a humidified incubator with 5% CO_2_ at 37°C as previously described [22].

### Expression Constructs and Stable Transduction

To generate glioblastoma cells with stable expressions of the full-length LRIG2 or the ectodomain of LRIG2, two lentiviral expression constructs, pLVX-puro-3×FLAG-LRIG2 and pLVX-puro-3×FLAG-LRIG2ecto, were generated by subcloning full-length LRIG2 (LRIG2) and ectodomain of LRIG2 (LRIG2ecto) from pEGFP-LRIG2 (gifted by Håkan Hedman) into pLVX-puro-3×FLAG. Lentiviral particles were produced by transfecting 293 T cells with pLVX-puro (Con), pLVX-puro-3×FLAG-LRIG2 (LRIG2) or pLVX-puro-3×FLAG-LRIG2ecto (LRIG2ecto) using Lenti-X lentiviral Expression Systems (Clontech) according to the manufacturer’s instructions. The glioblastoma cell lines U87 and U251 were infected with the respective lentivirus and selected with culture medium containing 1 µg/ml puromycin for 2 weeks. The stably transduced cell populations were pooled without clonal selection.

### Detection and elimination of Soluble LRIG2 Ectodomain in the Conditioned Medium

U87 glioblastoma cells stably transduced with control, full-length LRIG2 or LRIG2 ectodomain expressing vetor (2×10^6^ cells/group) were cultured in DMEM with 10% FBS for 48 h then maintained in 14 ml DMEM without FBS for another 48 h. The conditioned medium were collected and filtered through a 0.45 µm Millex filter (Millipore Corporation, USA). The filtered conditioned medium were subjected to Amicon Ultra-4 10 K centrifugal filter devices (Millipore Corporation, USA) spinning at 4,000×g for approximately 20 minutes to concentrate the proteins. The concentrated conditioned mediums were added with protease inhibitor (Sigma P8340) and 5× loading buffer, boiled at 100°C for 5 minutes and subjected to Western blotting for the detection of soluble LRIG2 ecdomain. The anti-Flag monoclonal antibody was used to detect the Flag-tagged proteins. To investigate the proliferative effects of soluble LRIG2 ectodomain in the conditioned medium on glioblastoma cells, the conditioned medium were filtered through a 0.45 µm Millex filter, followed by immunoprecipitation using Anti-Flag M2 Affinity Gel (Sigma A2220) to eliminate the soluble LRIG2 ectodomain as per the manufacturer’s instructions. The supernatant before immunoprecipitation and the resulting soluble LRIG2 ectodomain-free supernatant after immunoprecipitation were both used in the cell proliferation assay.

### Immunohistochemical Staining

The glioma samples of human and nude mice were fixed in a phosphate-buffered 4% formaldehyde solution and processed into paraffin blocks using standard methods. Paraffin sections with a thickness of 4 µm were incubated with anti-LRIG2 (1∶100), anti-Ki-67 (1∶200), anti-PCNA (1∶200), and anti-caspase3 (1∶100) antibodies at 4°C overnight as previously described [16]. Cytoplasmic immunoreactivity was scored artificially in four different categories: 0 for no or very faint immunoreactivity, 1 for weak immunoreactivity, 2 for moderate immunoreactivity, and 3 for intense immunoreactivity. Percentage scores were assigned as follows: <5%, 0; 5–25%, 1; 26–50%, 2; 51–75%, 3; >75%, 4.

### Immunofluorescence

Cells were fixed with 4% paraformaldehyde for 1 h at 4°C, permeabilized with 0.25% Triton X-100 for 5 min, blocked with 10% bovine serum albumin for 1 h and incubated with primary antibodies overnight at 4°C. After washing with PBS, cells were incubated with FITC or CY3-labeled secondary antibodies (1∶100, ProteinTech, USA) and subjected to immunofluorescence microscopy using appropriate filter.

### Quantitative RT-PCR

After cells were cultured with DMEM containing 10% FBS for 48 h, RNA isolation and quantitative RT-PCR were performed as described previously [24]. DNA primer sequences of LRIG2 and LRIG2ecto were designed as follows: sense, 5′-CAGTGCATAGCTGGAGGGAGTC-3′, anti-sense, 5′-TACAATGATGAGAAGC TGATTGGCTGCA-3′.

### Western Blotting Analysis and Immunoprecipitation

Western blotting analysis and immunoprecipitation were performed as described [24]. All the antibodies were diluted at optimal concentrations based on multiple experimental experiences.

### Cell Proliferation Assay

Cells were seeded into 96-well plate with 5×10^3^ cells in 200 µl suspension per well in triplicate and maintained in complete culture medium for 7 days. Every day, CCK8 was added and incubate for 2 h at 37°C. The absorbance of each well was measured with Microplate Reader using a wavelength of 450 nm.

### Soft Agar Colony Formation Assay

To perform anchorage-independent cell proliferation assay, six-well plates were first covered with a layer of 0.6% agar made in 10% FBS DMEM and 4×10^4^ cells per well were embedded in triplicates into 0.3% top agar gel containing DMEM supplemented with 10% FBS and incubated in a humidified chamber for 2 weeks. The six-well plates were inspected, and 30 randomly selected fields were photographed under a 10× microscopic lens and a 6× optical lens (60× magnification). Colonies were then counted, and the mean number of colonies per field was calculated. All experiments were done in triplicate.

### Flow Cytometry Method (FCM)-assessed Apoptosis

The extent of spontaneous apoptosis was determined with an Annexin V-FITC/Propidium Iodide Kit (KeyGEN Biotech, Nanjing, China) according to the manufacturer’s instructions as previously described [16].

### Measurement of Mitochondria Membrane Potential

Changes in the mitochondrial membrane potential were measured by flow cytometry using the mitochondrial membrane potential assay kit with JC-1 as described [25]. Briefly, the cells were collected after 48 h of incubation by trypsinization and centrifugation. The cell pellets were then washed twice with PBS. 0.5 ml JC-1 dye was added to the cell pellet and incubated at 37°C in dark. The staining solution was removed by centrifugation and cells were washed twice by JC-1 staining buffer. To assess the mitochondria membrane potential transition, cells were examined for each sample on an FL-1 versus FL-2 dot plot on a FACS Calibur and the data were analyzed using Cell Quest software (Becton Dickinson, USA).

### In Vivo Tumor Growth Study

Female 6- to 8- week-old BALB/c nude mice (n = 15) (Wuhan Laboratory Animal Center) were housed under specific pathogen-free conditions in a temperature- and humidity-controlled environment. All animal experiments were conducted in accordance with the Institutional Animal Care and Use Committee guidelines and the animal protocol used in this study was approved by local ethics committee (Ethical Committee of Tongji Hospital, Tongji Medical College, Huazhong University of Science and Technlogy, IRB ID: 2011A01). Briefly, U87 glioblastoma cells stably transduced with control vector, full-length LRIG2 or LRIG2 ectodomain were harvested and resuspended in PBS (2×10^7^/ml), respectively. A total of 2×10^6^ cells (in 100 µl of PBS) were injected subcutaneously into the right flank of each mouse (n = 5 per group). Tumor measures were taken every five days with calipers and volume was calculated as (width)^2^×(length)×(π/6). At the end of the experiment, mice were euthanized and tumors were surgically harvested, measured, and fixed in 4% paraformaldehyde overnight and analyzed by immunohistochemistry.

### Statistics

Statistical analyses were performed using SPSS 13.0 for Windows software (SPSS Inc., Chicago, IL, US). Significance between HGGs and LGGs in the immunohistochemical staining analysis was determined using chi-square test. Significance of others analyses was performed by One Way Anova followed by Bonferroni post-test. The level of statistical significance was defined as *P*<0.05.

## Results

### Expression of LRIG2 mRNA and protein positively correlates with the grade of glioma

**Figure 1:**
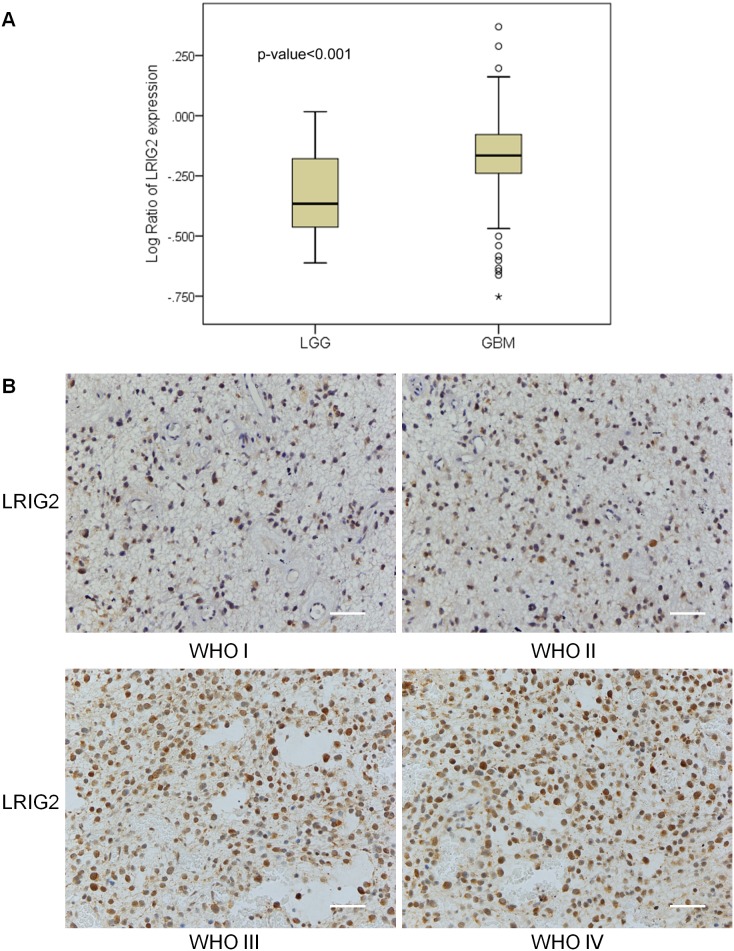
LRIG2 expression positively correlates with the WHO grade of glioma. (**A**) Microarray-based gene expression data of brain low grade gliomas (LGGs) and glioblastoma multiform (GBMs) were downloaded from public TCGA website. The expression level of LRIG2 gene in GBM was much higher compared with that in LGG (*P*<0.001). (**B**) Human gliomas of different grade were immunostained for the LRIG2 protein. Representative images of each grade of glioma were present (scale bar, 100 µm).

To investigate the expression levels of LRIG2 in different grade of glioma, we firstly used public TCGA data repositories as our primary source of samples. The level 1 data of gene expression, including 27 cases of LGGs and 466 cases of GBMs, were downloaded and analyzed. The results revealed that the LRIG2 gene expression level of GBM (WHO IV) was markedly higher than that of LGG (WHO I&II) (*P*<0.001) (Figure 1A). To further confirm the results, we collected the fresh human glioma samples of different grade to investigate the expression levels of LRIG2 proteins by immunnohistochemical staining. Of the investigated 20 LGGs (WHO I, II), the LRIG2 immunoreactivity (IR) of 14 cases were scored as 0 or 1 and 6 cases scored as 2 or 3. Of the 20 high-grade gliomas (HGGs, WHO III, IV), the LRIG2 IR of 5 cases were scored as 0 or 1 and 15 cases scored as 2 or 3 (Figure 1B). There was a significant difference in LRIG2 IR between low-grade gliomas and high-grade gliomas (*P* = 0.004), which indicated that the expression level of LRIG2 protein in HGGs was much higher compared to that in LGGs.

### Establishment of glioblastoma cell lines stably expressing the full-length LRIG2 and LRIG2 ectodomain

**Figure 2:**
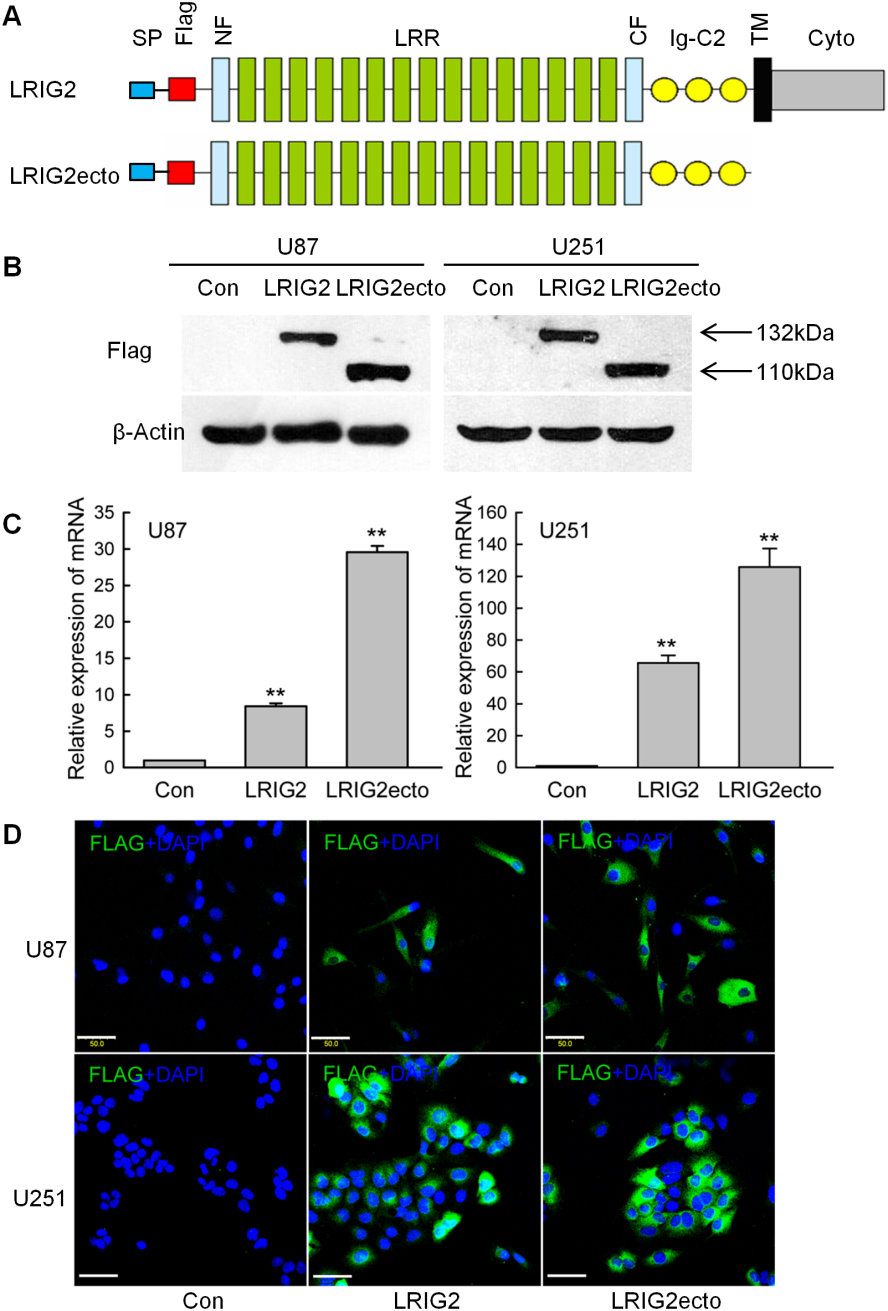
Establishment of glioblastoma cell lines stably expressing LRIG2 or LRIG2ecto. (**A**) Schematic drawing of the domain organization of the Flag tagged full-length LRIG2 and LRIG2 ectodomain. Indicated are the signal peptides (SP), Flag tag (Flag), the leucine-rich repeat domains, consisting of cysteine-rich N-flanking domain (NF), 15 leucine-rich repeats (LRR) and cysteine-rich C-flanking domain (CF), three immunoglobulin-like domains (Ig-C2), the transmembrane domain (TM) and the cytoplasmic tail (Cyto). (**B**) The total cell lysates of stably transduced cells, cultured in complete medium for 48 h, were subjected to immunoblotting using an anti-Flag antibody. β-Actin served as an internal loading control. (**C**) After maintained in complete medium for 48 h, the cells were subjected to total RNA extraction, followed by quantitative RT-PCR to measure the LRIG2 and LRIG2ecto mRNA expression levels. Expressions are shown as the fold changes of control cells (**P<0.01, vs con). (**D**) After cultured in complete medium for 48 h, stably transduced cells were subjected to immunofluorescence analysis. Flag staining is green, and nuclear stain is blue. Representative images of three independent experiments were shown. Scale bars, 50 µm.

LRIG1, which has been extensively studied, is proved to be a tumor suppressor [9] and the soluble LRIG1 ectodomain has also been found to act as a negative regulator of tumor growth [15]. To observe the effects of LRIG2 and LRIG2 ectodomain overexpression on glioblastoma growth *in vitro* and *in vivo* and elucidate the underlying mechanisms, we subcloned a human full-length LRIG2 cDNA and LRIG2 ectodomain cDNA into pLVX-puro-3×FLAG mammalian expression vectors respectively and transduced stably into U87 and U251 glioblastoma cells with pLVX-puro as the control vector. The domain organizations of the full-length LRIG2 and LRIG2 ectodomain were presented as Figure 2A. Figure 2B showed that the Flag-tagged full-length LRIG2 and LRIG2 ectodomain were successfully expressed. To assess the expression levels of corresponding mRNA, we assessed mRNA transcript levels in the stably transduced cells. LRIG2 and LRIG2ecto transduced U87 or U251 cells showed robustly increase in mRNA levels when compared with the corresponding control cells (Figure 2C). As assessed by immunofluorescence microscopy, Flag staining was observed in both cell lines transduced with LRIG2 and LRIG2 ectodomain (Figure 2D), which was in line with the results of western blotting. Taken together, glioblastoma cell lines stably expressing full-length LRIG2 and LRIG2 ectodomain were successfully established.

### The soluble LRIG2 ectodomain is released from glioblastoma cells and promotes the proliferation of glioblastoma cells

**Figure 3:**
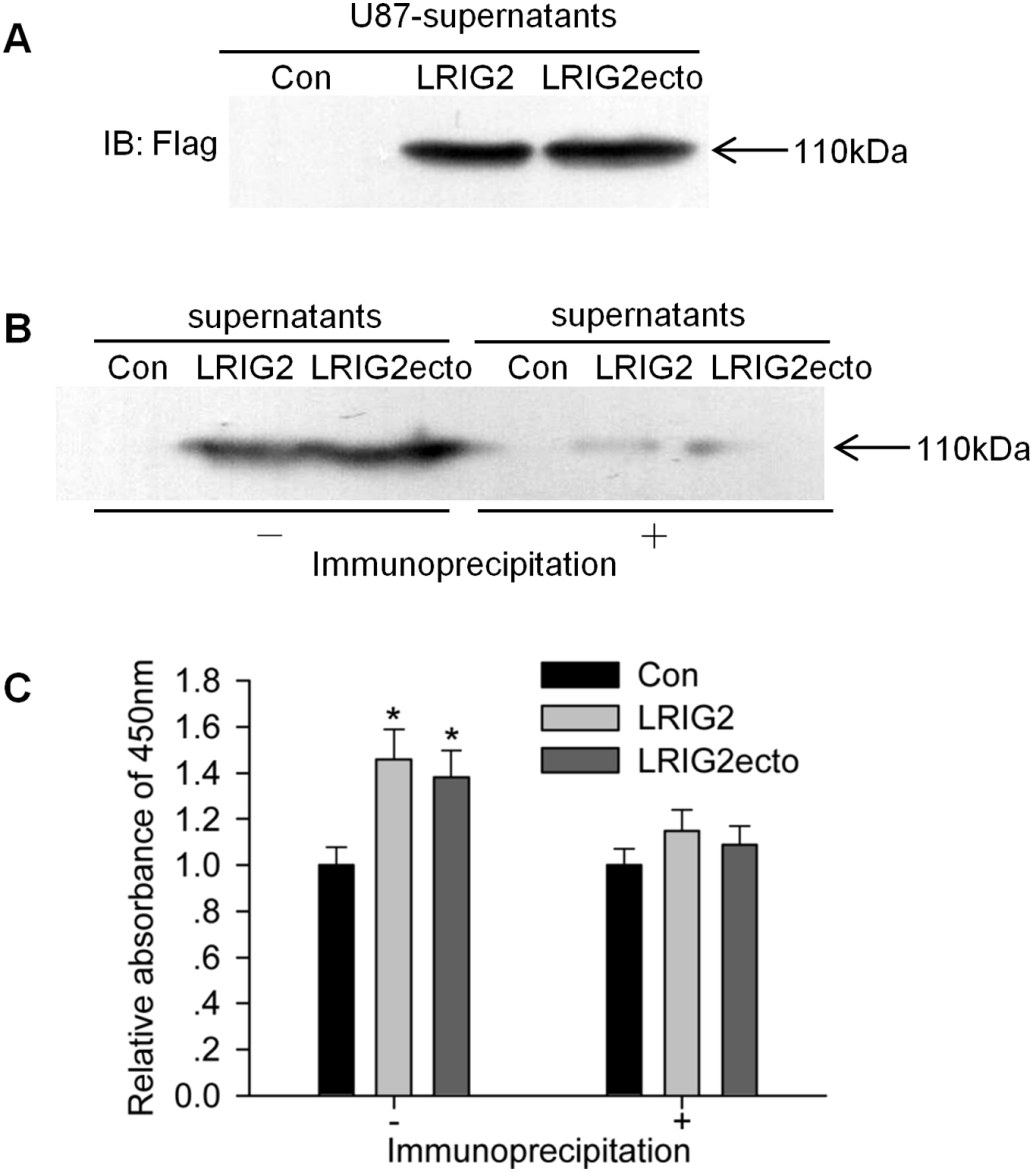
The detection of soluble LRIG2 ectodomain in the supernatants and its proliferative effects on glioblastoma cells. (**A**) Cells of U87-Con, U87-LRIG2 and U87-LRIG2ecto were cultured in DMEM for 48 h, the conditional cell culture supernatants were harvested, concentrated and subjected to western blot using an anti-Flag antibody. A representative blot from three independent experiments is shown. (**B**) After cells were cultured in DMEM for 48 h, the supernatants were harvested, filtered through 0.45 µm filter and subjected to immunoprecipitation to eliminate the expression of Flag-fusion proteins. The supernatants with or without immunoprecipitation were concentrated and subjected to western blotting. Representative image was shown. (**C**) The conditioned mediums after or before immnoprecipitation were subjected to proliferation assay. Statistical analysis was present (*P<0.05 vs con).

As previous study demonstrated that soluble LRIG1 ectodomains could be released from the full-length LRIG1 [14], we wanted to address whether soluble LRIG2 ectodomains could be released from glioblastoma cells. U87 glioblastoma cells were transduced with expression vectors encoding full-length LRIG2 carrying an N-terminal Flag epitope or LRIG2 ectodomain with a Flag tag respectively as illustrated in Figure 2A, and cultured in DMEM without FBS for 48 h. The conditioned cell culture supernatants were harvested, concentrated and subjected to western blotting using an anti-Flag antibody. Strikingly, the anti-Flag-reactive soluble protein was detected in the cell culture supernatants of cells with full-length LRIG2 overexpression, suggesting that the flag tagged protein fragment was released from the surface of glioblastoma cells. Strikingly, the molecular weight of the band corresponds to the predicted molecular weight of the entire LRIG2 ectodomain (Figure 3A). Consequently, we for the first time demonstrated that the soluble LRIG2 ectodomain was capable of being secreted by glioblastoma cells. Further, we evaluated the effects of the soluble LRIG2 ectodomain in the conditioned medium on the proliferation of glioblastoma cells. The results showed that the conditioned medium harvested from LRIG2 and LRIG2ecto overexpressing cells could significantly promote the proliferation of U87 cells (Figure 3C). After the Flag-fusion soluble LRIG2 ectodomain were down-regulated from the supernatants, the effects of conditioned medium on the proliferation of U87 cells were abrogated (Figure 3B, 3C), which indicated that the soluble LRIG2 ectodomain in the conditioned medium exerted proliferative effects on the glioblastoma cells. Collectively, we demonstrated for the first time that the soluble LRIG2 ectodomain could be released from the glioblastoma cells and promote the proliferation of glioblastoma cells *in vitro*.

### Full-length LRIG2 and LRIG2 ectodomain overexpressions promote the growth of glioblastoma cells *in vitro*

**Figure 4:**
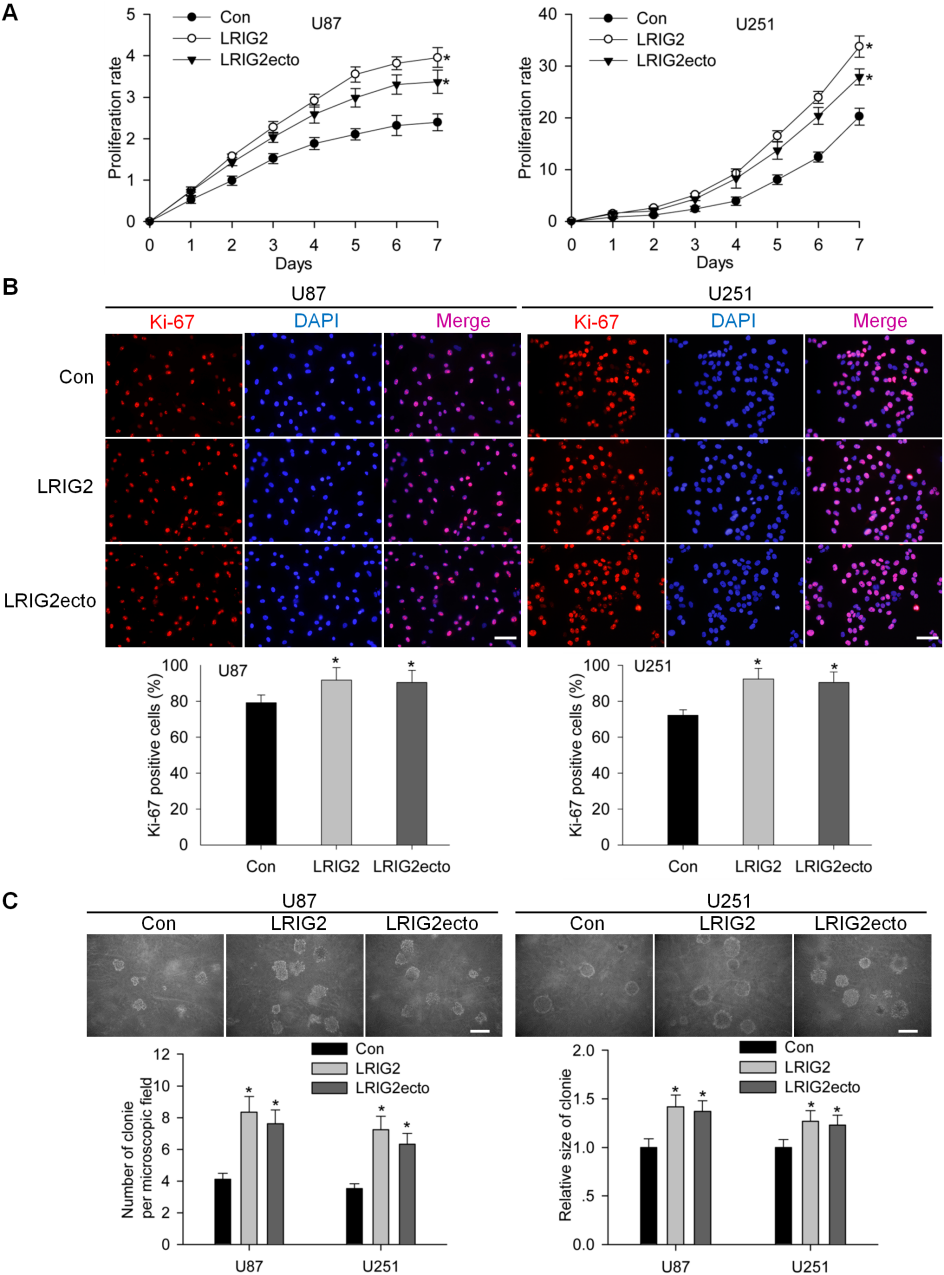
LRIG2 and LRIG2ecto overexpression promote the growth of glioblastoma cells *in vitro*. (**A**) Time-course effects of LRIG2 or LRIG2ecto overexpression on the growth of glioblastoma cells. The proliferation rates of cells were measured by CCK8 assay at the times indicated, which revealed that both LRIG2 and LRIG2ecto overexpression cells proliferate more rapidly than control cells (*P<0.05 vs con). (**B**) Representative images of immunofluorescent staining of Ki-67 were shown in the upper panel (scale bars, 50 µm). The percentages of Ki-67-positive cells in the LRIG2 and LRIG2ecto group were compared with that of the control group, respectively, shown in the lower panel (*P<0.05 vs con). (**C**) Soft agar colony formation assay was used to detect the anchorage-independent proliferation in the forementioned cells. The representative high-resolution images were shown (scale bars, 100 µm) and the quantitative analysis of colony numbers per microscopic field and colony sizes were present in the lower panel (compared with control, *P<0.05 vs con). All experiments were performed at least three times with consistent and repeatable results.

Previously, we demonstrated that overexpression of full-length LRIG1 inhibited the growth of glioblastoma cells *in vitro* [18], [26]. The soluble LRIG1 ectodomain was proved to inhibit the glioma cell proliferation recently [15]. However the effect of overexpression of full-length LRIG2 or LRIG2 ectodomain on the growth of glioblastoma cells has not been addressed yet. Firstly, we demonstrated that both LRIG2 and LRIG2ecto overexpressions markedly promoted the growth of U87 and U251 glioblastoma cells (Figure 4A). Secondly, we investigated the expression levels of Ki-67, a proliferation marker closely correlated with the proliferation of glioblastoma cells, in stably transduced U87 and U251 cells. The results revealed that the percentages of Ki-67 positive cells of LRIG2 and LRIG2ecto overexpressing U87 cells were (91.74±7.05)% and (90.59±6.72)% respectively, which were significantly higher than that of U87 control cells (79.19±4.29)% (*P*<0.05). In line with the results of U87 cells, the percentages of Ki-67 positive cells of LRIG2 and LRIG2ecto overexpressing U251 cells were also significantly increased compared to the U251 control cells (Figure 4B). Further, we investigated whether LRIG2 or LRIG2ecto altered glioblastoma anchorage-independent growth, a property that mimics tumorigenesis *in vivo*. Using soft agar colony formation assays, we found that gliobloastoma cells of LRIG2 or LRIG2ecto overexpression formed more and bigger colonies compared to the control cells (Figure 4C), indicating that overexpression of LRIG2 or LRIG2ecto enhances the ability of anchorage-independent growth. Taken together, these results revealed that full-length LRIG2 as well as LRIG2 ectodomain promoted the growth of glioblastoma cells *in vitro*.

### Full-length LRIG2 and LRIG2 ectodomain overexpressions inhibit the spontaneous apoptosis of glioblastoma cells through mitochondrial pathway *in vitro*

**Figure 5:**
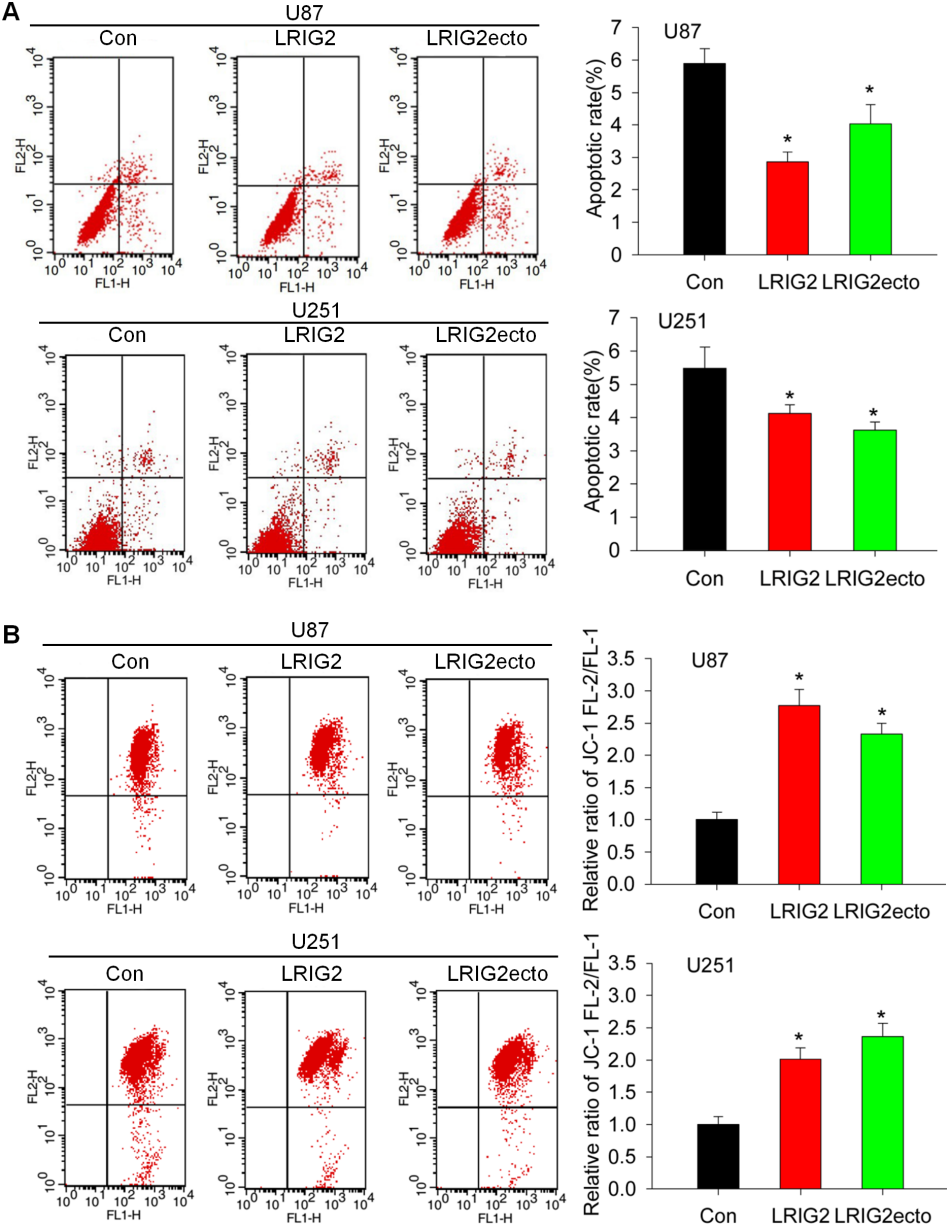
LRIG2 or LRIG2ecto overexpression inhibits the spontaneous apoptosis of glioblastoma cells *in vitro*. (**A**) Cells were cultured in DMEM with 10% FBS for 48 h, stained by Annexin V/PI and subjected to flow cytometry analysis. Early apoptotic populations were in the lower-right quadrant and late apoptotic cells were in the upper-right quadrant. Representative images were shown in the left panel and percentages of early and late apoptotic cells were expressed as the mean±SD of three independent experiments, shown in the right panel (*P<0.05 vs con). (**B**) Cells were maintained in complete medium for 48 h, stained with JC-1 and analyzed by flow cytometry. Representative flow cytometric images of mitochondrial membrane potential were shown. The shift of JC-1 fluorescence from red (FL2) to green (FL-1) indicates a collapse of the mitochondrial membrane potential. Change in mitochondrial membrane potential was determined by the ratio of JC-1 red fluorescence intensity to JC-1 green fluorescence intensity, shown in the right panel. Ratio was normalized to the control cells. (*P<0.05 vs con).

It has become apparent that tumor growth depends not only on the rate of cell proliferation but also on the rate of apoptosis [27]. Previously, we demonstrated that downregulation of LRIG2 expression by RNA interference increased spontaneous apoptosis of glioblastoma cells *in vitro* [22]. Congruently, by using flow cytometric analysis with Annexin V/PI staining, we revealed that the spontaneous apoptotic rates of LRIG2 overexpressing U87 and U251 cells were significantly decreased compared to the corresponding control cells (Figure 5A), indicating that LRIG2 overexpression inhibited the spontaneous apoptosis of glioblastoma cells. Most strikingly, we for the first time demonstrated that overexpression of LRIG2 ectodomain could also significantly decrease the spontaneous apoptotic rates of U87 and U251 glioblastoma cells (Figure 5A). To further explore the mechanisms of LRIG2 or LRIG2ecto mediated inhibition of apoptosis, we examined the mitochondrial membrane potential (MMP), depolarization of which is an early event in the mitochondrial pathway of apoptosis, using JC-1 red/green fluorescence measurement by flow cytometry [28]. The results revealed that the ratios of JC-1 FL-2/FL-1 of glioblastoma cells transduced with LRIG2 or LRIG2ecto were robustly increased compared to the corresponding control cells (Figure 5B), which indicated that LRIG2 and LRIG2ecto overexpressions both stabilized the MMP of glioblastoma cells. Collectively, we demonstrated that full-length LRIG2 and LRIG2 ectodomain overexpressions inhibited the spontaneous apoptosis of glioblastoma cells through mitochondrial pathway by stabilizing the mitochondria membrane potential.

### Full-length LRIG2 and LRIG2 ectodomain overexpressions promote the growth of tumor xenograft *in vivo*

**Figure 6:**
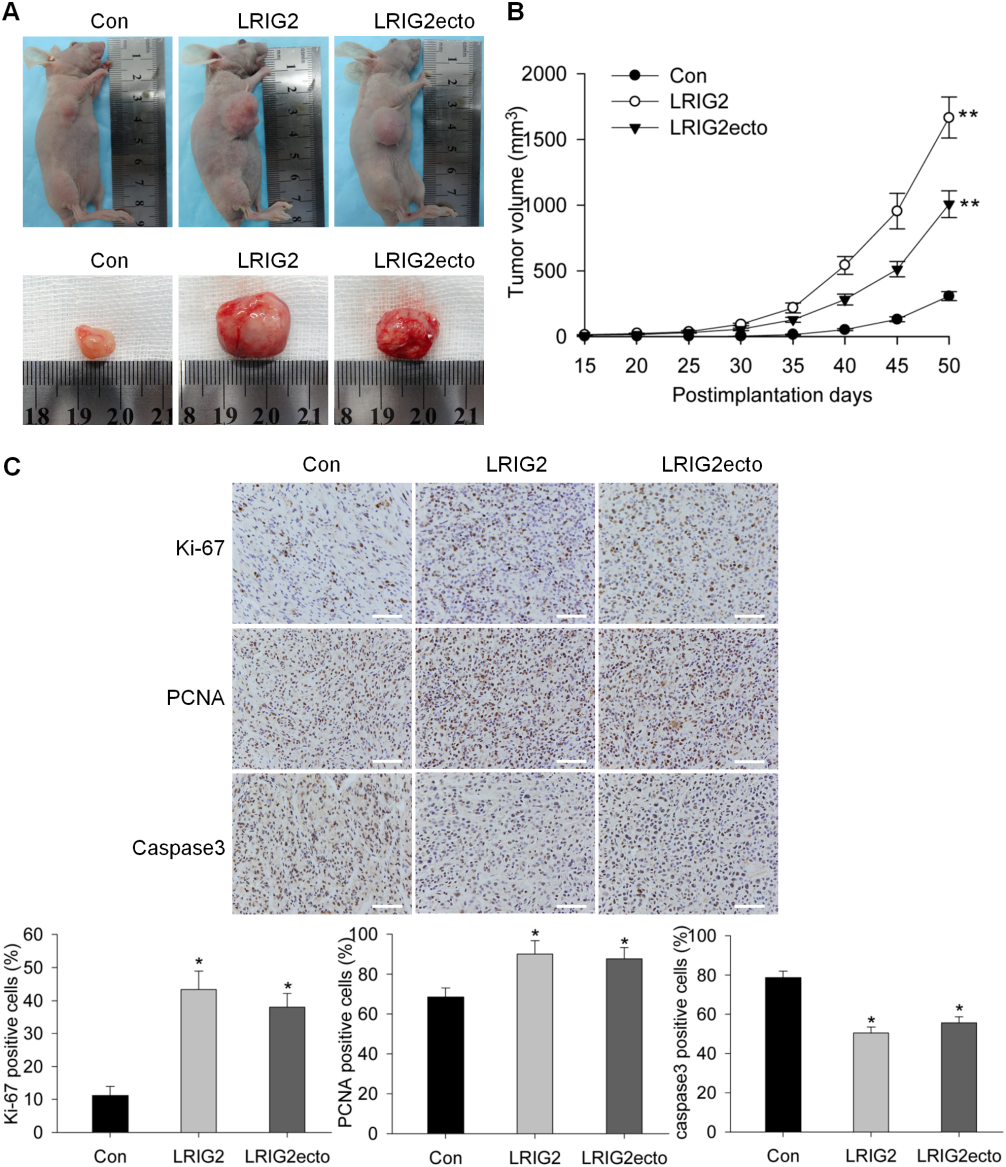
Overexpresson of LRIG2 or LRIG2ecto promotes tumor xenograft growth *in vivo*. (**A**) Photographs obtained on postimplantation Day 50 showing representative mice bearing U87-con, U87-LRIG2 and U87-LRIG2ecto xenografts. The corresponding surgically removed tumor xenografts were shown in the lower panel. (**B**) Tumor size was measured every five days from postimplantation day 15 and the growth curves of xenografts of each group were present (five mice per group). Data were shown as means±SD (**P<0.01 vs con). (**C**) The surgically removed tumor xenografts were fixed in 4% paraformaldehyde and subjected to immunohistochemistry staining of Ki-67, PCNA and Caspase3. The representative images were shown (scale bars, 100 µm). The percentages of immunostaining positive cells of Ki-67, PCNA and Caspase3 in the LRIG2 and LRIG2ecto group were compared with that of the control group, respectively, shown in the lower panel (*P<0.05 vs con).

We next determined whether the effects exerted by LRIG2 and LRIG2ecto overexpressions on the growth and apoptosis of glioblastoma cells *in vitro* could be demonstrated *in vivo*. Stably transduced glioblastoma cells were injected subcutaneously into the female 6- to 8- week-old nude mice and the tumor size of xenografts were measured every five days from postimplantation day 15. Consistent with the cell-based *in vitro* studies, tumor growth of LRIG2 and LRIG2ecto overexpression groups were strikingly promoted compared to the control group (Figure 6A, B) (**P<0.01). The xenografts were surgically removed and subjected to immunohistochemistry staining for Ki-67, PCNA, and caspase3. Similar to the results obtained from *in vitro* studies, the expression levels of Ki-67 and PCNA, two markers of cell proliferation activity, in LRIG2 and LRIG2ecto groups were both significantly increased and the levels of caspase3, a marker of apoptosis, were both markedly inhibited compared to the control group (Figure 6C). These results further demonstrated that LRIG2 and LRIG2ecto overexpressions promoted the growth of tumor xenograft by enhancing the proliferation and inhibiting the apoptosis *in vivo*.

### Full-length LRIG2 and LRIG2 ectodomain each can physically interact with EGFR in glioblastoma cells

**Figure 7:**
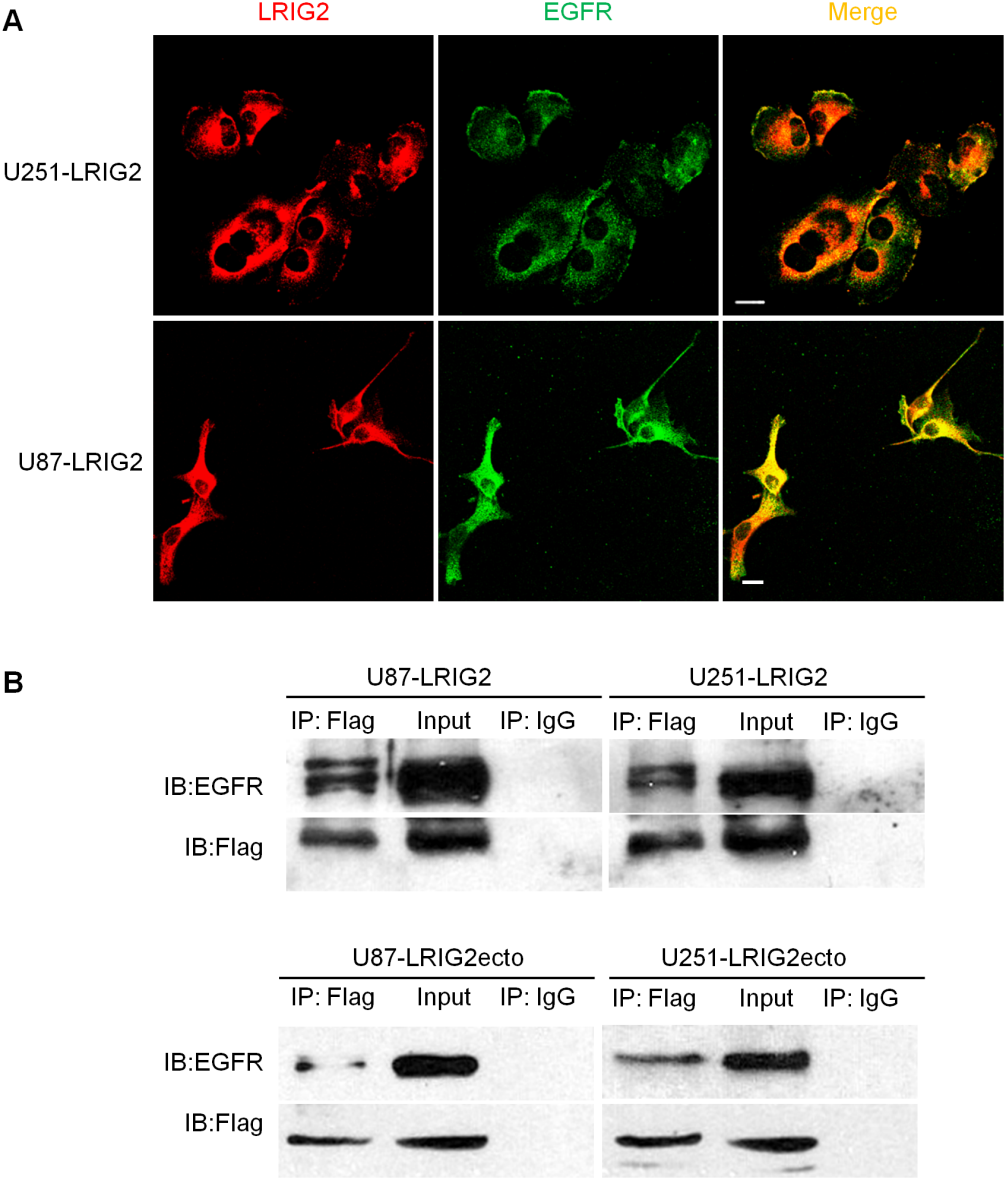
LRIG2 as well as LRIG2ecto interacts with EGFR in glioblastoma cells. (**A**) Confocal immunofluorescence laser microsopy showed colocalization of LRIG2 and EGFR in LRIG2 overexpresing U87 and U251 glioblastoma cells. Cells were stained with anti-LRIG2 and anti-EGFR antibodies, followed by using Cy3- and FITC-conjugated secondary antibodies. Yellow of merge panel indicates regions of colocalization. Scale bar, 50 µm. (**B**) Lysates from cells overexprssing LRIG2 or LRIG2ecto were immunoprecipitated with purified mouse anti-Flag monoclonal antibodies or control mouse IgG, and immunoprecipitates were blotted with antibodies to EGFR or Flag.

To explore the possible mechanisms underlying the forementioned effects of full-length LRIG2 and LRIG2 ectodomain on the glioblastomas, we examined whether LRIG2 or LRIG2ecto could interact with EGFR. Firstly, to determine the subcellular locations of full-length LRIG2 and possible sites of LRIG2-EGFR interaction, we examined the localization of LRIG2 and EGFR in LRIG2 overexpressing U251 and U87 cells by confocal immunofluorescence laser microscopy. As illustrated in Figure 7A, LRIG2 was found largely in the intracellular cytoplasm as well as in the plasma membrane and EGFR exhibited a similar distribution. Merging of images demonstrated that LRIG2 and EGFR signals overlap at the similar peripheral and intracellular locations, suggesting that these are sites of LRIG2-EGFR interaction. To further confirm the results, we precipitated Flag tagged LRIG2 from extracts of LRIG2-overexprssing cells using Flag antibody and detected the precipitates by western blotting using EGFR antibody. Consistent with the immunofluorescence results, this analysis revealed that LRIG2 physically interacted with EGFR (Figure 7B). Most strikingly, we found that the LRIG2 ectodomain was also capable of interacting physically with EGFR (Figure 7B), which indicates that the full-length LRIG2 interacts with EGFR through the recognition of the ectodomain of LRIG2 and EGFR.

### Full-length LRIG2 and LRIG2 ectodomain enhances EGFR activation and its downstream PI3K/Akt pathway

**Figure 8:**
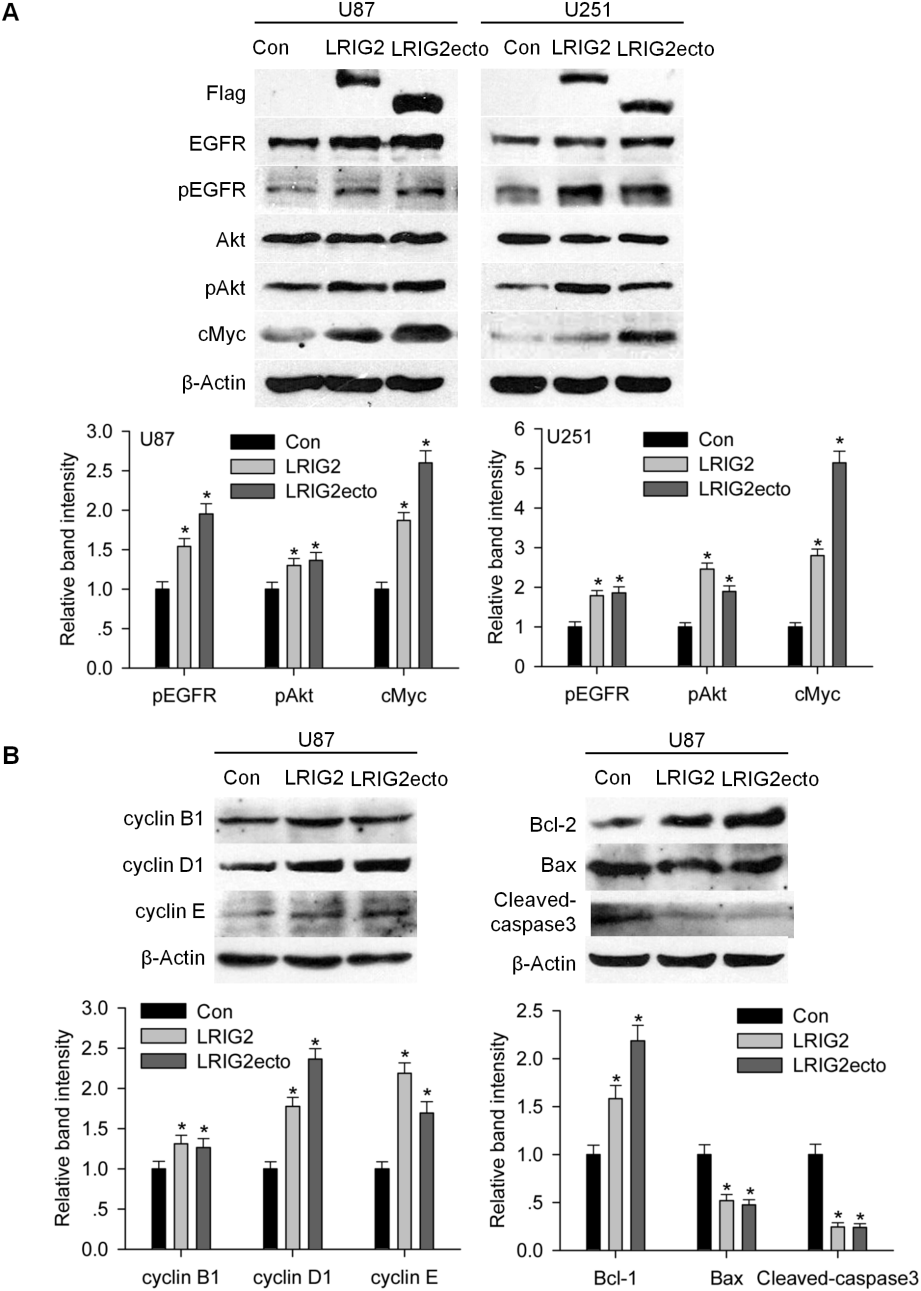
LRIG2 or LRIG2ecto overexpression enhances EGFR signaling and regulates the cell cycle and apoptosis related proteins. (**A**) After synchronization for 24 h, cells were cultured in DMEM with 10% FBS for 48 h and cell lysates were subjected to western blotting for the levels of EGFR signaling related proteins. Analysis of quantification of the bands intensity was shown in the lower panel (*P<0.05 vs con). (**B**) The indicated cells were starved for 24 h and cultured in complete medium for 48 h. The cell lysates were exacted and examined by western blotting for the cell cycle and apoptosis associated proteins. Analysis of quantification of the bands intensity was shown in the lower panel (*P<0.05 vs con).

**Figure 9:**
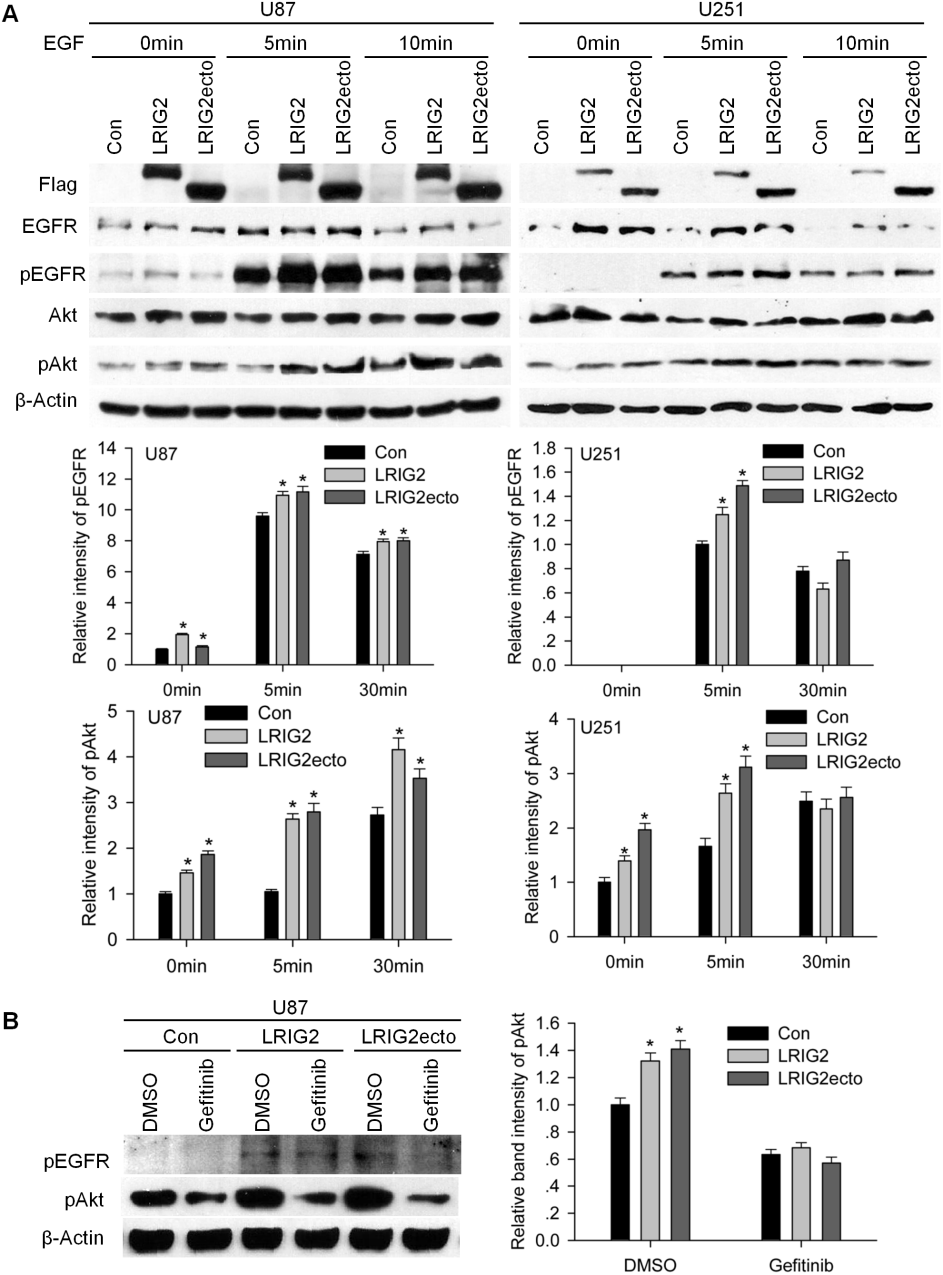
Overexpresson of LRIG2 or LRIG2ecto enhances the EGFR signaling in serum-free medium. (**A**) The stably tranduced U87 cells were synchronized in DMEM for 24 h and then stimulated with EGF (100 ng/ml) in serum-free medium for the times indicated. The expression levels of EGFR signaling proteins were analyzed by western blotting. The representative western blotting images of three independent experiments were shown and analysis of quantification of the bands intensity was shown in the lower panel (*P<0.05 vs con). (**B**) The stably tranduced U87 cells were cultured with 10% FBS addition with gefitinib (10 µM) or DMSO for 48 h. The total lysates were extracted and subjected to western blotting. The representative images of three independent experiments were present and quantification of the bands intensity was shown in the right panel (*P<0.05 vs con).

Based on the above results that LRIG2 and LRIG2ecto were capable of physically interacting with EGFR, we further explored the effects of LRIG2 and LRIG2ecto on the activation of EGFR and its downstream pathways. Stably transduced cells were cultured in DMEM with 10% FBS for 48 h, then the cell lysates were extracted and subjected to western blotting. The results revealed that the levels of phosphorylated EGFR (pEGFR) and phosphorylated Akt (pAkt) in LRIG2 and LRIG2ecto overexpressing cells were markedly increased compared to the corresponding control cells (Figure 8A). As expected, the expression levels of c-Myc, a transcription factor that triggers cell proliferation and regulated by PI3 K/Akt pathway [29], were also increased in LRIG2 and LRIG2ecto overexpressing cells (Figure 8A). The cell cycle proteins cyclin B1, cyclin D1, and cyclin E, which were closely associated with cell proliferation, were also expectedly enhanced. The anti-apoptotic protein Bcl-2 was increased and the pro-apoptotic proteins Bax and cleaved caspase3 were decreased (Figure 8B). To further confirm the effects of LRIG2 and LRIG2ecto on the activation of EGFR and the downstream PI3 K/Akt pathway, cells were cultured in DMEM without FBS for 24 h, followed by EGF stimulation. U87 cells overexpressing LRIG2 or LRIG2ecto exhibited not only increased levels of total and phosphorylated EGFR but also enhanced Akt activation with or without EGF stimulation (Figure 9A). U251 cells overexpressing LRIG2 or LRIG2ecto revealed strikingly increased levels of total EGFR with or without EGF stimulation, modestly increased phosphorylated EGFR and Akt activation with EGF stimulation for 5 minutes (Figure 9A). Next, we used gefitinib (10 µM) to inhibit the phosphorylation of EGFR followed by detecting the downstream PI3 K/Akt pathway and results revealed that inhibition of phosphorylation of EGFR abrogated the effects of LRIG2 and LRIG2ecto overexpressions on the Akt activation (Figure 9B), which further demonstrated that LRIG2 or LRIG2ecto overexpression enhanced the activation of PI3 K/Akt pathway through stabilizing the EGFR and enhancing the EGFR phosphorylation. Combining the forementioned results, we suggest that upregulation of LRIG2 or LRIG2ecto lead to stabilization of EGFR and activation of EGFR, thereby enhancing the downstream PI3K/AKT pathway.

## Discussion

In the present study, we revealed that the expression levels of LRIG2 transcript in glioblastomas and LRIG2 protein in HGGs were both significantly higher compared to that in LGGs and we for the first time demonstrated that LRIG2 expression levels positively correlated with the grade of glioma, which predicted that LRIG2 might serve as a negative prognostic factor associated with poor glioblastoma survival. This mirrors to some extent the situation in oligodendrogliomas [20] and early-stage squamous cell carcinoma of the uterine cervix [21], where high expression of LRIG2 is associated with poor survival, whereas LRIG1 expression negatively correlates with tumor grade [10], [11], [12] and associates with better survival in numerous tumors [11], [12], indicating that the functions of LRIG2 and LRIG1 may be different in the progression of tumors.

To further confirm the role of LRIG2 in glioblastoma and explore the possible underlying mechanisms, we established glioblastoma cells with stable expressions of the full-length LRIG2. In line with the forementioned results, we showed that overexpression of full-length LRIG2 promoted the proliferation and inhibited the apoptosis of glioblastoma cells *in vitro* and *in vivo*. Combined with our previous reported finding that downregulation of LRIG2 inhibits glioblastoma cell growth *in vitro* [22], our results provided compelling evidences in support of the proposal that full-length LRIG2 served as a tumor promoter in glioblastoma. Congruently, it is recently reported that, by using an animal model of PDGF-induced glioma, the Lrig2E12−/− mice showed increased spontaneous mortality, reduced growth rate and protection against PDGFB-induced glioma and Lrig2E12+/+ mice developed gliomas at a higher frequency and of higher malignancy than Lrig2E12−/− mice, which indicated a pro-tumoregenic role of Lrig2 in the progression of oligodentroglioma. [19]. Collectively, data to date suggest that LRIG2 in human and Lrig2 in mouse both play critical roles in the genesis and progression of glioma. In previous study, we demonstrated that LRIG1 inhibited the proliferation and promoted the apoptosis of glioblastoma cells in *vitro* and *in vivo* [18], [26], suggesting that LRIG1 exerts as a tumor suppressor in glioblastoma, which is in contrast to the results of LRIG2 herein presented. However, the mechanism underlying the function difference between LRIG2 and LRIG1 remain largely unexplored.

LRIG proteins share the same domain organization: an ectodomain consisting of 15 leucine-rich repeats (LRR) with cysteine-rich N- and C-terminal flanking domains and three immunoglobulin-like domains, a transmembrane domain and a cytoplasmic tail [4]. It has been reported that a recombinant fragment of the LRIG1 ectodomain causes growth inhibition of a panel of carcinoma cells [30]. Noteworthy, soluble LRIG1 ectodomain has recently been demonstrated to be shed naturally from the full-length LRIG1 and suppress cell proliferation [14]. Moreover, this soluble LRIG1 ectodomain has been shown to inhibit the genesis and progression of glioma *in vivo* [15]. However, little is known regarding the functions of LRIG2 ectodomain. In the present study, we for the first time demonstrated that the soluble LRIG2 ectodomain was capable of being released from glioblastoma cells and soluble LRIG2 ectodomain promoted the proliferation and inhibited the apoptosis of glioblastoma cells *in vitro* and *in vivo* in a similar manner to the full-length LRIG2, which suggested that LRIG2 ectodomain played a similar role to the full-length LRIG2 in the progression of glioblastoma. The herein present function of LRIG2 ectodomain in progression of glioblastoma was opposite to the full-length LRIG1 [18], [26] and LRIG1 ectodomain [14], [30], while in line with full-length LRIG2, which leads to the proposal that LRIG2 ectodomain may contribute to the function difference of the full-length LRIG2 and full-length LRIG1 in glioblastoma progression. To our knowledge, the soluble counterparts of transmembrane proteins can be released mainly by two distinct mechanisms: proteolytic processing and microvesicle shedding [31]. Soluble LRIG1 ectodomain has been demonstrated to be secreted from the full-length LRIG1 through the proteolytic processing [14], the mechanism underlying the release of the soluble LRIG2 ectodomain is not addressed and deserves further study.

Further, in order to provide more compelling evidence to support our proposal, we explored the possible mechanisms underlying the effects of full-length LRIG2 and LRIG2 ectodomain on the proliferation and apoptosis of glioblastoma. Accumulating evidence indicates that LRIG1 functions as a tumor suppressor in humans by negatively regulating tyrosine kinase receptors of the EGFR family [9], [32]. The mechanisms by which LRIG1 restricts growth factor signaling include three different ways, such as enhancing liand-induced EGFR ubiquitylation and degradation [5], [6], promoting Met degradation in a lysosome-dependent and cbl-independent manner [7], and inhibiting binding of GDNF with Ret [8]. Nevertheless, LRIG1 ectodomain has also been demonstrated to suppress EGF signaling without any apparent downregulation of EGFR levels [14], [30]. It is not known yet that whether or not the full-length LRIG2 and LRIG2 ectodomain exert the similar roles in the regulation of EGFR signaling. Remarkably, in the present study we for the first time demonstrated that full-length LRIG2 and LRIG2 ectodomain both could physically interact with EGFR, increase the level of EGFR and enhance the activation of EGFR and its downstream PI3K/Akt pathway, resulting in increment of pro-proliferative and anti-apoptotic proteins and attenuation of pro-apoptotic proteins. Besides the EGFR signaling, the PDGF-induced immediate-early gene induction of Fos and Egr2 has also recently been demonstrated to be regulated by Lrig2 in primary MEF cells. However, Lrig2 just regulated PDGF-induced immediate-early gene induction and had no effects on the PDGFR protein levels or phosphorylation events of PDGFR, Akt in MEF cells [19]. The tumor promoting effects of LRIG2 on receptor tyrosine kinases (RTKs) might be prominent in tumor cells and subtle in non-tumor cells. Most notably, the results herein present suggest that LRIG2 ectodomain plays a similar role to the full-length LRIG2 in the regulation of EGFR signaling pathways, whereas LRIG1 and LRIG1 ectodomain exert the opposite functions. Such observations further strengthen the proposal that LRIG2 ectodomain may be responsible for the function difference of LRIG2 and LRIG1 in the progression of glioblastoma.

Based on our finding that soluble LRIG2 ectodomain is capable of being released from full-length LRIG2, it is reasonable to speculate that the soluble LRIG2 ectodomain can release from the glioblastoma cells as an effective pro-growth factor in the microenvironment, interact with EGFR in an autocrine or paracrine way and enhance the EGFR signaling to promote the growth of glioblastoma. The amino acid sequence of human LRIG2 was 47% identical to human LRIG1, whereas the ectodomains of LRIG2 and LRIG1 were highly conserved [33]. Therefore the mechanisms underlying the function differences between LRIG2 ectodomain and LRIG1 ectodomain in the progression of glioblastoma are needed to be further addressed in the future.

Taken together, we report for the first time that LRIG2 expression levels positively correlate with the grade of glioma. Most strikingly, we demonstrate for the first time that the soluble LRIG2 ectodomain is capable of being released from glioblastoma cells. Both full-length LRIG2 and LRIG2 ectodomain potently promote the proliferation and inhibited the apoptosis of glioblastoma cells *in vitro* and *in vivo* through physically interacting with EGFR, stabilizing EGFR, and enhancing EGFR activation and its downstream PI3 K/Akt pathway. Going forward, it will be important to determine how LRIG2 ectodomain is released from glioblastoma cells, whether this kind of release is universal, and how LRIG2 ectodomain interact with EGFR and enhance the activation of EGFR. We believe that LRIG2 ectodomain may provide a promising therapeutic target in the treatment of glioblastoma multiform in the future.
